# The Southern Dental Association’s Proceedings

**Published:** 1896-07

**Authors:** 


### The Southern Dental Association's Proceedings.

The volume of transactions of the last meeting of this body has just come to hand. It consists of 185 pages, and was published by the S. S. White Dental Manufacturing Company. That is a sufficient guarantee as to the mechanical execution of the work. In looking over it we find it filled with good things which no dentist who wishes to keep abreast of the times can afford to miss; so we say to all such, get a copy and make the good things your own, and if you will do that it will not be necessary to specify further here. It is unfortunate that an index was omitted.

